# The Alternative TrkAIII Splice Variant, a Targetable Oncogenic Participant in Human Cutaneous Malignant Melanoma

**DOI:** 10.3390/cells12020237

**Published:** 2023-01-05

**Authors:** Lucia Cappabianca, Veronica Zelli, Cristina Pellegrini, Michela Sebastiano, Rita Maccarone, Marco Clementi, Alessandro Chiominto, Pierdomenico Ruggeri, Ludovica Cardelli, Marianna Ruggieri, Maddalena Sbaffone, Maria-Concetta Fargnoli, Stefano Guadagni, Antonietta R. Farina, Andrew R. Mackay

**Affiliations:** 1 Department of Biotechnological and Applied Clinical Sciences, University of L’Aquila, 67100 L’Aquila, Italy; 2 Department of Dermatology, University of L’Aquila, 67100 L’Aquila, Italy; 3 Department of Pathology, Saint Salvatory Hospital, 67100 L’Aquila, Italy

**Keywords:** cutaneous malignant melanoma, TrkA, alternative TrkA splicing, TrkAIII, reductive stress, unfolded protein response, Xbp-1, oncogene activation mechanism, A375 cells, lestaurtinib, entrectinib

## Abstract

Post-therapeutic relapse, poor survival rates and increasing incidence justify the search for novel therapeutic targets and strategies in cutaneous malignant melanoma (CMM). Within this context, a potential oncogenic role for TrkA in CMM is suggested by reports of *NTRK1* amplification, enhanced TrkA expression and intracellular TrkA activation associated with poor prognosis. TrkA, however, exhibits tumour-suppressing properties in melanoma cell lines and has recently been reported not to be associated with CMM progression. To better understand these contradictions, we present the first analysis of potential oncogenic alternative TrkA mRNA splicing, associated with TrkA immunoreactivity, in CMMs, and compare the behaviour of fully spliced TrkA and the alternative TrkAIII splice variant in *BRAF(V600E)*-mutated A375 melanoma cells. Alternative TrkA splicing in CMMs was associated with unfolded protein response (UPR) activation. Of the several alternative TrkA mRNA splice variants detected, TrkAIII was the only variant with an open reading frame and, therefore, oncogenic potential. TrkAIII expression was more frequent in metastatic CMMs, predominated over fully spliced TrkA mRNA expression in ≈50% and was invariably linked to intracellular phosphorylated TrkA immunoreactivity. Phosphorylated TrkA species resembling TrkAIII were also detected in metastatic CMM extracts. In A375 cells, reductive stress induced UPR activation and promoted TrkAIII expression and, in transient transfectants, promoted TrkAIII and Akt phosphorylation, enhancing resistance to reductive stress-induced death, which was prevented by lestaurtinib and entrectinib. In contrast, fully spliced TrkA was dysfunctional in A375 cells. The data identify fully spliced TrkA dysfunction as a novel mechanism for reducing melanoma suppression, support a causal relationship between reductive stress, UPR activation, alternative TrkAIII splicing and TrkAIII activation and characterise a targetable oncogenic pro-survival role for TrkAIII in CMM.

## 1. Introduction

Cutaneous malignant melanomas (CMM) are increasing in frequency and, despite improvements in treatment, exhibit frequent post-therapeutic relapse and continue to carry a poor prognosis, justifying the search for new therapeutic targets and strategies [1,2,3]. In addition to known mutation-activated oncogenic signalling pathways [4], recent attention has focused on a potential oncogenic role for the neurotrophin receptor tropomyosin-related tyrosine kinase A (TrkA) in CMM. TrkA mediates melanocyte and melanoma cell responses to neurotrophic factors [5,6], the gene that encodes TrkA, *NTRK1*, is frequently amplified in CMMs, increased TrkA expression and intracellular activation have been positively correlated with CMM progression and poor outcome [7,8,9,10] and TrkA fusion oncogenes have been detected in CMMs and spitzoid melanomas [11,12]. However, this possibility is contradicted by reports that TrkA inhibits melanoma cell proliferation and promotes differentiation, and it has been recently reported not to be associated with CMM metastatic progression, consistent with melanoma-suppressing potential [7,13]. This controversy is reminiscent of similar reports of TrkA’s tumour-suppressing and oncogenic roles in neuroblastoma (NB) and NB cell lines [14,15,16,17]. In NB, this has been explained by stress-regulated alternative TrkAIII splicing that can convert tumour-suppressing signals from fully spliced TrkA into oncogenic signals from the alternative TrkAIII splice variant [14,15], as an alternative oncogenic mechanism to *NTRK1* amplification and chimeric TrkA fusion. Alternative TrkAIII splicing has also recently been reported in Merkel cell polyomavirus (MCPyV)-positive Merkel cell carcinomas in association with advanced-stage metastatic disease and post-therapeutic relapse [18,19]. 

The alternative TrkAIII mRNA splice variant (GeneBank OP866787) is characterised by *NTRK1* exons 6, 7 and 9 skipping and results in expression of a structurally compromised receptor devoid of the extracellular TrkA D4 immunoglobulin-like domain. This domain is required for fully spliced TrkA cell surface expression and prevents spontaneous receptor activation [14,15,20,21]. As a consequence, TrkAIII is not expressed at the cell surface but accumulates in pre-Golgi membranes, where it exhibits spontaneous activation [14,15]. This results in signalling through the phosphoinositol-3-kinase/protein kinase B/nuclear factor kappa-binding (PI3K/Akt/NF-κB) but not the RAS/mitogen-activated kinase (MAPK) pathway, which differs to signalling from neurotrophin-activated cell surface fully spliced TrkA though Ras/MAPK [14,15]. This difference underpins TrkAIII oncogenic activity in NB cells, which is characterised by pro-survival (B-cell lymphoma extra-large, Bcl-xL, myeloid cell differentiation protein-1, Mcl-1 and superoxide dismutase-2, SOD-2) and pro-angiogenic (vascular endothelial cell growth factor, VEGF, matrix metallpproteinase-9, MMP-9) gene expression, centrosome amplification and stress-regulated metabolic adaptation, within a de-differentiated cancer-stem-cell-like context [14,15,22,23]. TrkA signalling through RAS/MAPK, on the other hand, inhibits NB cell proliferation, promotes neural differentiation and suppresses angiogenesis and tumorigenicity in NB models [14,15,17]. *NTRK1*, therefore, produces a wider range of TrkA protein isoforms than previously considered, with tumour-suppressing and oncogenic potential [14,15,24,25], and TrkAIII represents the pathological equivalent of a previously reported engineered oncogenic TrkA D4-deletion mutant [19].

In NB cells, alternative TrkAIII splicing is promoted by hypoxia, nutrient deprivation and reductive stress, in association with activation of the unfolded protein response (UPR) [14,15,26]. These conditions are common in solid tumours, including CMMs, and promote the selection of resistant tumour subpopulations through transcriptional and post-transcriptional mechanisms, including alternative splicing [26,27,28,29,30,31,32,33,34,35,36]. In this study, therefore, we assessed whether stress-regulated alternative TrkA mRNA splicing, resulting in the expression of potential oncogenic TrkA splice variants, may explain the controversy surrounding TrkA involvement in CMM, in addition to providing novel potential therapeutic targets.

## 2. Materials and Methods

### 2.1. Patients and CMM Tissues

Tissues from CMM patients (11 females and 19 males) diagnosed in the Departments of Dermatology and Surgery, University of L’Aquila, Italy, with ages ranging from 35 to 92 years old, were assessed in this study (Table S1). Written consent was obtained from all patients, and the study was approved by L’Aquila University Ethics committee (protocol number 50/2018.19). RNAs were purified from 8 snap-frozen primary CMMs (patients P.1–8), 19 snap-frozen metastatic CMMs (patients P.9–27), formalin-fixed paraffin-embedded (FFPE) tissues corresponding to snap-frozen CMMs (patients P.1–7, P.9–15, P.18–24, P.26 and P.27), paired FFPE primary and metastatic CMM tissues from 3 patients (patients 28–30), for which snap-frozen tissues were not available, and 2 uninvolved skin samples. Protein extracts for Western blotting were prepared from 3 snap-frozen metastatic CMMs (patients P.9, P.18 and P.19). For indirect immunofluorescence (IF), 5 μm FFPE tissue sections from 26 CMM patients were analysed, comprising 7 primary CMMs and 19 CMM metastases (patients P.1–7, P.9–15, P.18–24 and P.26–30). FFPE tissues for patients P.8, P.16, P.17 and P.25 were not available for indirect IF. 

### 2.2. Antibodies, Reagents and Cell Lines

Rabbit polyclonal and mouse monoclonal anti-human TrkA carboxyl-terminus (C14 and B3, 200 μg/mL) antibodies were from Santa Cruz Biotechnology (Dallas, TX, USA). Rabbit monoclonal anti-human Y490-phosphorylated TrkA antibody (9141, 36 μg/mL), rabbit polyclonal Akt (9272, 31 μg/mL) and phosphorylated Phospho-Ser 473-Akt (4060, 91 μg/mL) antibodies were from Cell Signaling Technology (Danvers, MA, USA). Mouse monoclonal anti-β-actin antibody (AC-15) was from Merk (Darmstadt, GE). Secondary antibodies included horseradish peroxide (HRP)-conjugated goat anti-rabbit and rabbit anti-mouse antibodies (1 mg/mL) from Bethyl Laboratories Inc (Fortis, Waltham, MA, USA) and Alexa Flour 488-labeled donkey anti-rabbit and Alexa Fluor donkey anti-mouse antibodies from Life Technologies (1 mg/mL) (Fortis, Waltham, MA, USA). The Prolong^TM^ Gold anti-fade reagent with DAPI was from Invitrogen (Thermo-Fisher Scientific, Waltham, MA, USA). Sigma*FAST* Protease inhibitor tablets (S8820) were from Sigma-Aldrich (St. Louis, MO, USA). The multitarget tyrosine kinase inhibitor lestaurtinib [37] and the Alk/Trk inhibitor entrectinib [38] were from Merck (Darmstadt, GE). Dithiotreitol (DTT) was from Sigma-Aldrich (St. Louis, MO, USA). The A375 melanoma cell line was obtained from the American Type Culture Collection (ATCC CRL-1619, Manassas, VA, USA) and was confirmed as carrying the B-rapidly accelerated fibrosarcoma serine/threonine kinase (*BRAF*) *V600E* mutation and wild-type *NRas* by PCR sequencing of purified genomic DNA (not shown). Cells were cultured in high-glucose Dulbecco’s modified Eagle’s medium (DMEM), supplemented with 10% heat-inactivated foetal calf serum (FCS), glutamine, penicillin and streptomycin. Stable TrkA and TrkAIII SH-SY5Y transfectants have been described previously [14] and were cultured in complete DMEM culture medium, supplemented with zeocin (Thermo Fischer, Milan, Italy). 

### 2.3. Transient A375 Transfections

A375 melanoma cells were grown to 70–80% confluence in complete medium in Corning 100 mm cell culture dishes (Merk, Milan, Italy) for protein and RNA extraction and Corning 96 well flat-bottom cell culture plates (Merck, Milan, Italy) for Incucyte automated live-cell analysis (Sartorius, Goettigen, Germany). Supercoiled plasmid pcDNA3.1 TrkA or pcDNA3.1 TrkAIII (6 μg) was transfected into cells using the TransIT-X2^TM^ Dynamic Delivery System, as directed by the manufacturer (Mirus Bio, Madison, WI, USA). Briefly, TransIT-X2 transfection reagent and plasmid (pcDNA3.1-TrkA or pcDNA3.1-TrkAIII) were mixed at a ratio of 3:1 in Opti-MEM serum-free medium and incubated at room temperature for 15 min. After incubation, the TransIt–plasmid mixture was added drop-wise to cells, and cells were cultured for a further 48 h in complete medium to ensure sufficient protein expression. For Incucyte assays, transient 48 h A375 transfectants in 96-well culture plates were analysed using Incucyte Cell Green cytotoxicity assays over a 48 h time course, in the presence or absence of DTT (5 mM), lestaurtinib (100 nM) or entrectinib (100 nM). For RNA and protein analyses, 48 h transient A375 transfectants were incubated for the times indicated, in the presence or absence of DTT (5 mM), lestaurtinib (100 nM) or entrectinib (100 nM), prior to RNA and protein extractions.

### 2.4. In Vitro Incucyte Cytotoxicity Assays

Cytotoxicity was assessed over a 48 h time course in an IncuCyte^®^ S3 Live-Cell Analysis System incubator, as directed (Sartorius, Goettingen, Germany), using Incucyte^®^ Cytotox Green Dye (4633) for detecting cytotoxic disruption of cell membrane integrity. Briefly, transient A375 transfectants at ≈80% confluence in 96-well plates were treated in the presence of 150 nM Incucyte^®^ Cytotox Green Dye and the presence or absence of 5 mM DTT, 100 nM lestaurtinib or 100 nM entrectinib. Incucyte analysis software was programmed for time-lapse photography of 2 independent areas per well, at 2 h intervals and 10× magnification. Cell behaviour was analysed by time-lapse video, and cell death evaluated as the mean ± s.e. percentage of cells exhibiting nuclear Incucyte^®^ Cytotox Green Dye uptake, counted directly in phase contrast micrographs at 12 h intervals in 3 independent experiments, each performed in duplicate. 

### 2.5. RNA Extraction and Reverse Transcriptase Polymerase Chain Reaction (RT-PCR) Analysis

Surgical tissues specimens placed in RNAse-free tubes were immediately snap-frozen in liquid nitrogen and stored at −80 °C, until use. Melanin-rich CMM regions were selected macroscopically and macro-dissected into 3 mm^2^ pieces in order to minimise contamination from non-melanoma cutaneous tissues, and then pulverised in a Tissue Lyser LT (Quiagen, Milan, Italy) at 5 oscillations per second for 2 min, and RNAs were purified from the lysis buffer provided in the Quick-RNA^TM^ Miniprep Kit, as described by the manufacturer (Zymo Research, Freiberg im Breisgau, GE). RNA purity and concentrations were evaluated in a nanodrop spectrophotometer, as directed (Thermo Fisher Scientific, CA, USA). Purified RNAs were reverse-transcribed using a Superscript IV reverse transcription kit, as directed (Thermo Fischer Scientific, Waltham, MA, USA), and reverse transcription reactions, at various dilutions, were subjected to RT-PCR. For 18S rRNA, 1 μL of 1:1000 (0.05 ng) RT dilutions was subjected to RT-PCRs consisting of 35 cycles of 30 s at 94 °C, 30 s at 58 ° and 30 s at 72 °C, using the primer set 5′-AAACGGCTACCACATCCAAG-3′ and 5′-CCTCGAAAGAGTCCTGTATTG-3′. For TrkA exons 1–8, 1 μL of non-diluted RT (50 ng) was subjected to RT-PCRs consisting of 35 cycles of 60 s at 94 °C, 30 s at 68 °C and 1 m at 72 °C, using the primer set 5′-ATGCTGCGAGGCGGACGGCGC-3′ and 5′-GGAGGCCTGGCCGAAGGGGTT-3′. For TrkA exons 8–17, 1 μL of non-diluted RT (50 ng) was subjected to RT-PCRs consisting of 35 cycles of 60 s at 94 °C, 30 s at 65 °C and 1 m at 72 °C, using the primer set 5′-AACCCCTTCGGCCAGGCCTCC-3′ and 5′-CTAGCCCAGGACATCCAGGTA-3′ (1298 bp product). For the fully spliced TrkA exon 5/6 splice junction, 1 μL of non-diluted RT (50 ng) was subjected to RT-PCRs consisting of 35 cycles of 30 s at 94 °C, 30 s at 61 °C and 30 s at 72 °C, using the primer set 5′-GGTGATGAAATCTGGGGGTCT-3′and 5′-TTGACCTGAACAGAGACCTCTGC-3′ (132 bp product). For TrkAIII-specific exon 5–8 splice junction, 1 μL of non-diluted RT (50 ng) was subjected to RT-PCRs consisting of 35 cycles of 30 s at 94 °C, 30 s at 60 °C and 30 s at 72 °C, using the primer set 5′-AATGCCAGCTGTGTCCCG-3′ and 5′-TGGTCTCATTGAGCACGGAG-3′ (139 bp product). For unconventional Xbp-1 splicing, 1 μL of non-diluted RT (50 ng) was subjected to RT-PCRs consisting of 35 cycles of 40 s at 95 °C, 30 s at 59 °C and 1 m at 72 °C, using the primer set 5′-TTACGAGAGAAAACTCATGGC-3′ and 5′-CGGTCCAAGTTGTCCAGAATGC-3′. RT-PCRs were performed in duplicate and repeated. For densitometric analysis, 1.5% agarose gels were digitally photographed and images analysed by Image J software (ImageJ bundled with Java 1.8.0_172), with inter-gel comparisons performed using common 18S rRNA RT-PCR product and DNA ladder standards, where appropriate. 

### 2.6. DNA Sequencing

TrkA exon 1–8 and exon 8–17 RT-PCR products were purified from ethidium bromide-stained agarose gels using a Jet Quick gel extraction spin kit, as directed (Genomed, Harrow, UK), cleaned using a EuroSAP PCR enzymatic Clean-Up kit, as directed (Euroclone, Milan, Italy) and PCR amplified using the TrkA exons 1–8 primer set 5′-ATGCTGCGAGGCGGACGGCGC-3′ and 5′-GGAGGCCTGGCCGAAGGGGTT-3′ or the TrkA exons 8–17 primer set 5′-AACCCCTTCGGCCAGGCCTCC-3′ and 5′-CTAGCCCAGGACATCCAGGTA-3′, using a BigDye Terminator V.2.1. Cycle Sequencing kit, as directed (Thermo-Fisher Scientific, CA, USA). Re-amplified products were then sequenced in a mono-capillary DNA sequencer (ABI PRISM 310, Thermo-Fischer Scientific, CA, USA) via double-stranded Sanger sequencing. 

### 2.7. Protein Extraction and Western Blotting

Surgical tissue specimens placed in RNAse-free tubes were immediately snap-frozen in liquid nitrogen and stored at −80 °C. Melanin-rich regions of CMM tissues were selected macroscopically and macro-dissected into 3 mm^2^ pieces in order to minimise contamination from non-melanoma cutaneous tissues. Frozen tissues were then pulverised in a Tissue Lyser LT (Quiagen, Milan, Italy), at 5 oscillations per second for 3 min, and proteins were extracted in lysis buffer (PBS containing 0.5% sodium deoxycholate, 1% NP40, 0.1% SDS, 1 mM sodium orthovanadate, 1 mM PMSF, 1 μg/mL of pepstatin A and protease inhibitor cocktail (SigmaFAST Protease inhibitors, Sigma-Aldrich, St Louis, MO, USA) containing AEBSF, EDTA, Bestatin, E-64, leupeptin and aprotinin, on ice, as previously described [18]. Protein concentrations were evaluated by Bradford assay, as directed (BioRad, Milan, Italy). 

Protein extracts, at the concentrations indicated in figure descriptions, were separated by reducing SDS-PAGE, transferred to nitrocellulose blotting membranes (Hybond, Amersham, Amersham, UK) and analysed for immunoreactivity to either rabbit polyclonal anti-human TrkA carboxyl terminus (C14, 1:1000 dilution) or mouse monoclonal anti-human TrkA carboxyl terminus (B3, 1:1000 dilution), rabbit monoclonal anti-human Y490-phosphorylated TrkA (pY490-TrkA, 1:1000 dilution), mouse monoclonal anti-human β-actin (1:1000 dilution) antibodies or pre-immune IgG, with protein loading also assessed by Ponceau S staining (Merck, Darmstadt, Germany). Western blots were incubated with primary antibodies overnight at 4 °C, washed extensively in PBS containing 0.1% Tween 20, and then incubated with appropriate secondary antibodies for 1 h at room temperature. Western blots were repeated a minimum of two times.

### 2.8. Indirect IF

FFPE sections (5 μm) were de-paraffinized, re-hydrated and processed for antigen retrieval by incubation in 0.01 M sodium citrate buffer (pH 6.0) for 20 min at 98 °C. Sections were blocked in blocking solution (1x PBS, BSA 1%, Tx100 0.03%), incubated overnight at 4 °C with rabbit polyclonal anti-human TrkA (C14, 1:100 dilution in blocking solution) or rabbit monoclonal anti-human Y490-phopsphorylated TrkA (pY490-TrkA, 1:100 dilution, in blocking solution) primary antibodies, washed extensively in PBS, and then incubated with appropriate fluorochrome-conjugated Alexa Fluor secondary antibodies (diluted 1:1000 in blocking solution) for 2h at room temperature. Slides were then washed, mounted in Prolong^TM^ Gold anti-fade reagent with DAPI (Thermo-Fisher Scientific, Waltham, MA, USA) and visually scored by scanning confocal microscopy (Leica TCS SP5 II). For cells, A375 cells cultured on Lab-Tek glass chamber slides (MERK, Darmstadt GE) were rinsed in PBS, fixed for 15 min in 4% formaldehyde at 37°C, and then permeabilised for 20 min in methanol at −20°C. Fixed permeabilised cells were then rehydrated, blocked in blocking solution (1x PBS, BSA 1%, Tx100 0.03%) and incubated for 2 h with either rabbit polyclonal anti-human TrkA (C14, 1:100 dilution in blocking solution) or mouse monoclonal anti-human TrkA (B3, 1:100 dilution in blocking solution) combined with rabbit monoclonal anti-human Y490-phosphorylated TrkA (pY490-TrkA, 1:100 dilution in blocking solution). Following incubation, cells were washed extensively in PBS and incubated with appropriate fluorochrome-conjugated secondary antibody (1:200 dilutions in blocking solution) for 2 h at room temperature, mounted in Prolong^TM^ Gold anti-fade reagent with DAPI (Thermo-Fisher Scientific, Waltham, MA, USA) and analysed by scanning confocal microscopy (Leica TCS SP5 II).

### 2.9. Statistical Analysis 

Data were analysed by Student’s *t*-test (https://www.graphpad.com/quickcalcs/ttest1.cfm), accessed on several occasions from November to December 2022 and statistical significance was associated with probabilities of ≤0.05.

## 3. Results

### 3.1. Alternative TrkA mRNA Splicing in CMM Tissues

**Figure 1:**
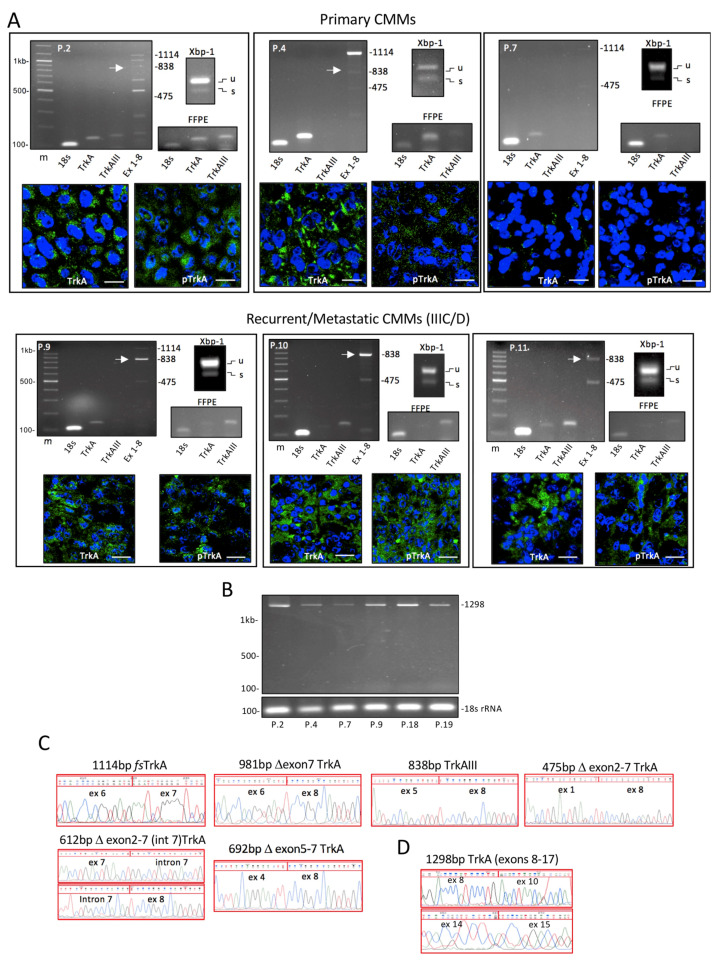
Alternative TrkA mRNA splicing, unconventional Xbp-1 splicing and TrkA and phosphorylated TrkA isoforms in CMMs. (**A**) Representative RT-PCRs, demonstrating 18S rRNA, TrkA, TrkAIII, TrkA exon 1–8 splice (Ex 1–8) alternative splice variants, and unspliced (u) and unconventionally spliced Xbp1 (s) RT-PCR products, in RNAs from 3 snap-frozen primary (P.2, P.4 and P.7) and 3 snap-frozen metastatic CMMs (P.9, P.10 and P.11), and in corresponding formalin-fixed paraffin-embedded (FFPE) tissues (m = DNA markers). Accompanying IF micrographs demonstrate immunoreactivity for TrkA, using the rabbit polyclonal anti-TrkA (C14) antibody (TrkA, green), and for phosphorylated TrkA, using the rabbit monoclonal anti-phosphorylated Y490 TrkA (9141) antibody (pTrkA, green), with DAPI-stained nuclei (blue), in each CMM (bar = 10 μm). (**B**) RT-PCRs demonstrating 18S rRNA and TrkA exon 8–17 RT-PCR products in a representative selection of CMMs. (**C**) DNA sequences of RT-PCR products generated by TrkA exon 1–8 primer sets, confirming the presence of splice junction sequences consistent with fully spliced (*fs*) TrkA, Δexon 7 TrkA, TrkAIII, Δexon 2–7 TrkA, Δexon 2–7(int 7) TrkA and Δexon 5–7 TrkA (Δ = skipped exon(s), and (**D**) TrkA sequence generated by TrkA exon 8–17 primer sets in the single product detected in CMMs, exhibiting exon 9 skipping consistent with the TrkAI isoform.

RT-PCR using primers spanning TrkA exons 1 to 8 detected products corresponding to fully spliced TrkA (1114 bp) and 5 alternative TrkA splice variants of 981 bp, 838 bp, 692 bp, 612 bp and 475 bp in RNAs from 8 snap-frozen primary CMMs and 19 snap-frozen metastatic CMMs. RT-PCR using primers spanning TrkA exons 8 to 17 detected a single 1298 bp sequence characterised fully spliced TrkA product in all TrkA exon 1–8 RT-PCR-positive CMMs (Figure 1, Figures S1 and S2). 

The fully spliced 1114 bp TrkA exon 1–8 RT-PCR product was detected in all 8 (100%) primary CMMs and in 11 of 19 (57.9%) CMM metastases, representing ≈70% of all snap-frozen CMM tissues, and was also detected in 2 uninvolved skin samples. Relatively high 1114 bp fully spliced TrkA to 18S rRNA RT-PCR ratios characterised 3 of 8 (37.5%) primary and 5 of 19 (26.3%) metastatic CMMs, moderate to low ratios characterised 5 of 8 (62.5%) primary and 8 of 19 (42.1%) metastatic CMMs, and the 1114 bp TrkA RT-PCR product was not detected in 6 of 19 (31.6%) metastatic CMMs. Sequence-characterised alternative splice variants detected included: 981 bp Δexon 7 TrkA in 1 of 8 (12.5%) primary CMMs and 2 of 19 (10.5%) metastatic CMMs; 838 bp TrkAIII in 3 of 8 (37.5%) primary CMMs and 12 of 19 (63.2%) metastatic CMMs; 692 bp Δexon 5–7 TrkA in 1 of 8 (12.5%) primary CMMs and 2 of 19 (10.5%) metastatic CMMs; 612 bp Δexon 2–7(int7) TrkA, with partial intron 7 inclusion, in 1 of 19 (5.3%) metastatic CMMs; and 475 bp Δexon 2–7 TrkA in 5 of 8 (62.5%) primary CMMs and 11 of 19 (57.9%) metastatic CMMs (Figure 1, Table 1 and Supplementary Figures S1, S2 and S4).

Online sequence analyses (https://www.ncbi.nlm.nih.gov/orffinder/), accessed on various occasions from January to November 2022, confirmed that TrkAIII was the only open reading frame alternative TrkA splice variant expressed in CMM tissues. All other variants (Δexon 7 TrkA, Δexon 5–7 TrkA, Δexon 2–7(Int 7) TrkA and Δexon 2–7 TrkA) contained frame shifts resulting in early stop codons down-stream of the novel splice junctions.

**Figure 2:**
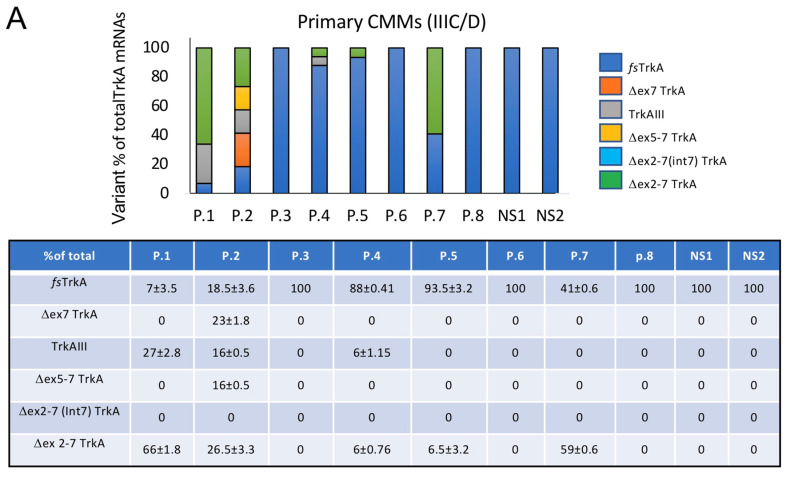

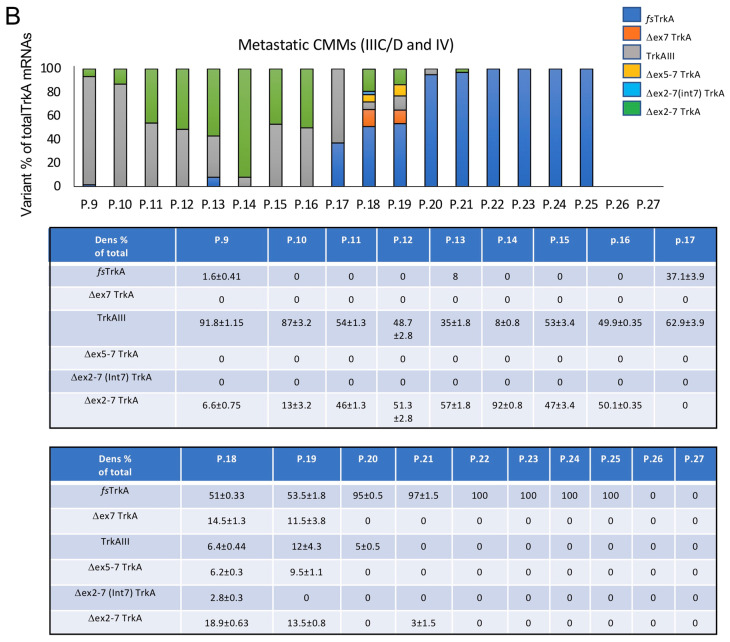
Alternative TrkA mRNA splicing ranges from exclusive fully spliced TrkA to almost exclusive TrkAIII expression in CMMs. Histograms and accompanying tables demonstrating differences in fully spliced (*fs*) TrkA, Δexon7 TrkA, TrkAIII, Δexon 5–7TrkA, Δexon 2–7(Int 7)TrkA and Δexon 2–7TrkA RT-PCR products, as densitometric mean ± s.e. percentages of all variants in: (**A**) 8 snap-frozen primary CMMs (upper histogram and table) and (**B**) 19 snap-frozen metastatic CMMs (lower histogram and table). The distribution of TrkA splice variants in histograms is enumerated in the accompanying tables, as the mean ± s.e. of each individual variant as a percentage (%) of all variants in each individual patient, in duplicate RT-PCRs repeated twice (see Supplementary Figures S1, S2 and S4, for all RT-PCRs).

In densitometric analyses, fully spliced TrkA, as a percentage of total TrkA RT-PCR products, ranged from 100% (P.6, P.8 and P.22–25) to 0 or almost 0 (P.9–P.16). TrkAIII expression also ranged from 92% (P.9) and 87% (P.10) to 0 in primary CMMs (P.3 and P.5–8) and metastatic CMMs (P.21–27) (Figure 2, Figures S1, S2 and S4). Densitometric ratios of fully spliced TrkA to TrkAIII, representing the only mRNAs with in-frame coding sequences capable of producing isoforms with intact tyrosine kinase domains, revealed predominant TrkAIII over fully spliced TrkA expression in 1 of 8 (12.5%) primary CMMs (patient P1) and 9 of 19 (47.4%) metastatic CMMs (patients P.9–17). Fully spliced TrkA predominated over TrkAIII RT-PCR in 7 of 8 (87.5%) (patients P.2–8) primary CMMs and 8 of 19 (42.1%) metastatic CMMs (patients P.18–25). Fully spliced TrkA and TrkAIII were not detected in two metastatic CMMs (patients P.26 and P.27) (Figure 2). With respect to *BRAF* mutational status, TrkAIII mRNA expression was detected in 3 of 6 (50%) primary *BRAF* wild-type CMMs (P.2, P.4 and P.28) and in 8 of 12 (66.6%) metastatic *BRAF* wild-type CMMs (P.9–12, P.17–19 and P.28), and in 3 of 4 (75%) *BRAF*-mutated primary CMMs (P.1, P.29 and P.30) and in 6 of 9 (66.6%) *BRAF*-mutated metastatic CMMs (P.13–16, P.29 and P.30) (Figure 1 and Figure 2, and Supplementary Table S1, Figures S1 and S4).

TrkA 132 bp exons 6/7 junction RT-PCR products were detected in all snap-frozen primary and metastatic 1114 bp TrkA exon 1–8 RT-PCR-positive CMMs in corresponding FFPE tissues, and also in the paired primary (a) and metastatic (b) FFPE CMM tissues (patients P.28–30), for which snap-frozen tissues were not available (Figure 1, Table 1 and Supplementary Figures S1–S3). TrkAIII-specific 139 bp exon 5/8 splice junction products were detected in all 838 bp TrkAIII RT-PCR-positive snap-frozen CMMs, in corresponding FFPE tissues, and also in the paired primary (a) and metastatic (b) FFPE CMM tissues (patients P.28–30) (Figure 1, Table 1 and Supplementary Figures S1–S3). RT-PCR for 132 bp TrkA and 139 bp TrkAIII products were not assessed in patients P.8, P.16, P.17 and P.25 due to limited RNA. These data confirm the feasibility of detecting TrkAIII in FFPE CMM tissues.

The nonsense splice variant Δexon 2–7 TrkA was predominant in 2 of 8 (25%) primary CMMs and 4 of 19 (≈21%) metastatic CMMs (Figure 1 and Figure 2, and Supplementary Figure S1). Detection of this variant suggests suppression of nonsense-mediated RNA decay [39,40], augmenting the possibility of generating a mutant protein or exhibiting long non-coding RNA activity [41,42,43].

Unconventional Xbp-1 splicing, as an index of UPR activation [44,45], was detected in 4 of 5 snap-frozen primary CMMs (4 primary CMMs P.5–8 were not analysed due to limited RNA) and in 16 of 16 (100%) of metastatic CMMs (patients P.16, P.17 and P.25 were not assessed due to limited RNA). Unconventional Xbp-1 splicing was not detected in uninvolved skin samples (Figure 1, and Supplementary Figures S1–S3).

These data demonstrate that alternative TrkA mRNA splicing occurs in CMMs in frequent association with UPR activation and intracellular TrkA and phosphorylated TrkA isoform expression. The data also demonstrate that the alternative TrkAIII mRNA splice variant predominates over fully spliced TrkA mRNA expression in ≈50% of metastatic CMMs and is the only in-frame alternative splice variant expressed by CMMs.

### 3.2. Indirect IF Analyses

TrkA isoform immunoreactivity was detected in 9 of 10 primary CMMs (90%) analysed (patients P.1–6 and P.28–30). P.7′s primary CMM tissue was negative for TrkA immunoreactivity and patient P.8′s primary CMM tissue was not available for IF. TrkA immunoreactivity was also detected in 16 of the 19 (84.2%) metastatic CMMs analysed (patients P.9–15, P.18–23 and P.28–30) and was not detected in P.24′s, P.25′s or P.27′s metastatic CMM tissues, and P.16′s, P.17′s and P.25′s metastatic CMM tissues were not available for IF (Figure 1, Table 1 and Supplementary Figures S1–S3).

Intracellular phosphorylated TrkA IF immunoreactivity was clearly detected in 5 of 10 (50%) primary CMMs (patients P.1, P.2 and P.28a–30a), was barely detectable in 2 primary CMMs (patients P.4 and P6) and was not detected in 3 primary CMMs (patients P.3, P.5 and P.7). In metastatic CMMs, intracellular phosphorylated TrkA IF immunoreactivity was clearly detected in 15 of 19 (≈79%) metastatic CMMs (patients P.9–13, P.15, P.18–21, P.23 and P.28b–30b), was barely detectable in 2 metastatic CMMs (patients P.14 and P.22) and was not detected in 3 metastatic CMMs (patients P.24, P.26 and P.27) (Figure 1, Table 1 and Supplementary Figures S1–S3). P.16′s, P.17′s and P.25′s metastatic CMM tissues were not available for IF.

This section summarises the frequent association detected between intracellular TrkA and phosphorylated TrkA isoform immunoreactivity, alternative TrkAIII mRNA splicing and UPR activation in the majority of the metastatic CMMs.

### 3.3. TrkA Isoform(s) Detected in CMM Extracts by Western Blotting

**Figure 3:**
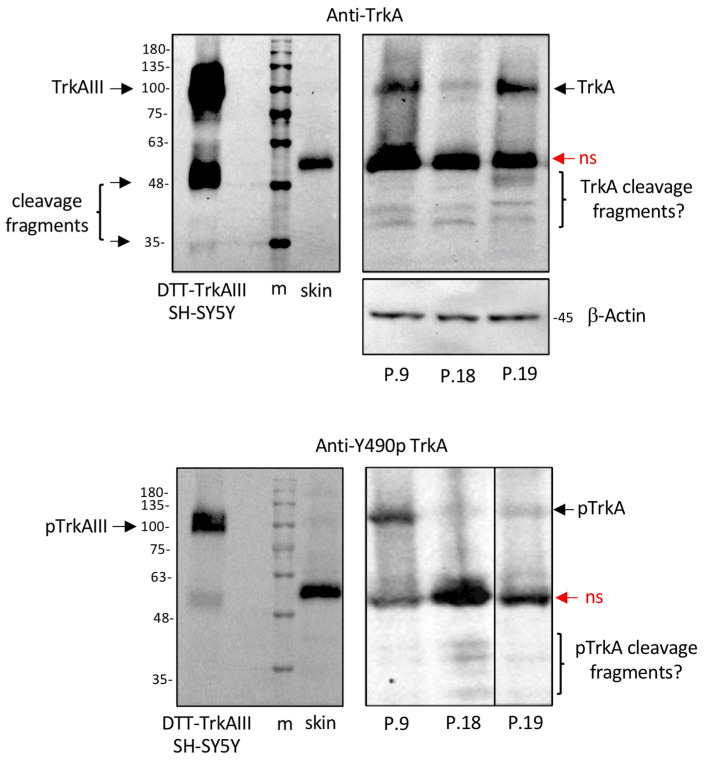
Metastatic CMMs express phosphorylated TrkA isoforms that resemble TrkAIII. Western blots demonstrating specific immunoreactive TrkA and phosphorylated TrkA species (pTrkAIII/pTrkA) (arrows) and non-specific species (ns), in stable TrkAIII SH-SY5Y transfectant extracts treated with DTT (5 mM for 6 h) (10 μg), in a normal skin extract and in extracts from 3 metastatic CMMs (100 μg). Potential TrkA and phosphorylated TrkA cleavage products are also indicated (cleavage fragments?).

Specific 100 kDa TrkA and phosphorylated TrkA immunoreactive species were detected in protein extracts from three metastatic CMMs, exhibiting TrkAIII mRNA expression (patients P.9, P.18 and P.19). These species exhibited similar electrophoretic mobility to 100 kDa TrkAIII expressed in stable transfected SH-SY5Y cells. Additional smaller (<48 kDa) specific immunoreactive species were also detected in these metastatic CMM extracts (Figure 3).

The data demonstrate the presence of a phosphorylated TrkA protein isoform, consistent with TrkAIII, in protein extracts from snap-frozen metastatic CMM tissue samples.

### 3.4. TrkAIII Expression in A375 Cells

**Figure 4:**
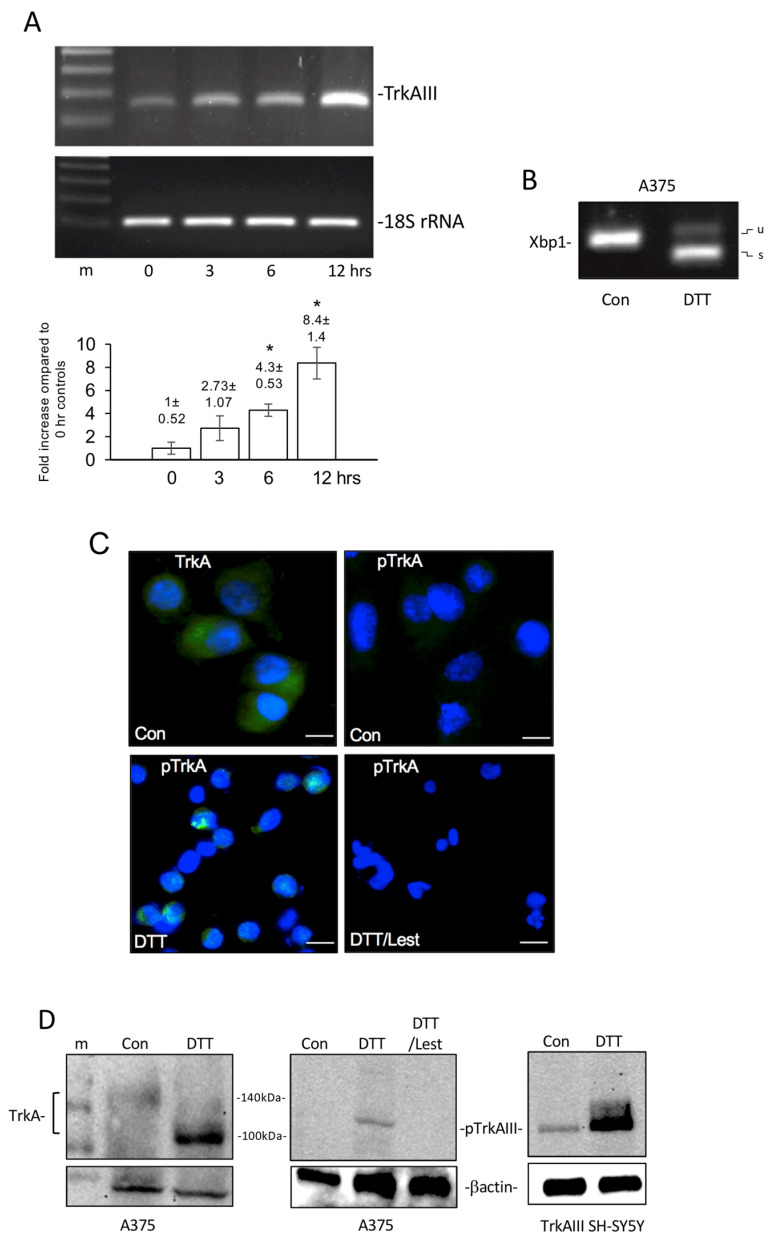
DTT induces UPR activation and promotes TrkAIII mRNA expression and TrkA isoform activation in A375 melanoma cells. (**A**) RT-PCR using TrkAIII specific exon 5/8 junction primers and accompanying histogram, demonstrating a significant (*) increase in A375 cell TrkAIII mRNA expression, following treatment with DTT for 3, 6 and 12 h. Histogram results are expressed as the mean ± s.e. densitometric fold increase in TrkAIII expression, relative to 18S rRNA, compared to 0 h controls (1 fold), in 3 independent experiments. (**B**) RT-PCR demonstrating induction of unconventional Xbp-1 splicing in A375 cells following a 6 h DTT treatment (u = unspliced and s = spliced Xbp1). (**C**) Micrographs demonstrating TrkA (TrkA, green) but not phosphorylated TrkA immunoreactivity (pTrkA, green) (upper 2 panels) in untreated A375 cells, and the induction of phosphorylated TrkA immunoreactivity (pTrkA, green) following 6 h DTT treatment in the absence (DTT) but not presence of 1 μM lestaurtinib (DTT/Lest) (bar = 10 μm). (**D**) Western blots demonstrating low ≈ 140 kDa TrkA immunoreactivity in untreated A375 cell extracts (Con) (100 μg) and induction of a ≈100 kDa TrkA isoform in extracts from A375 cells following 6 h DTT treatment (left panels, 100 μg), and the induction of a phosphorylated ≈100 kDa TrkA isoform, in extracts of A375 cells following 6 h DTT treatment, not detected in untreated A375 cell extracts (Con), or in extracts from A375 cells following a 6 h treatment with DTT in the presence of 1 μM lestaurtinib (DTT/Lest) (middle panels, 100 μg). Right-hand panels demonstrate constitutive (Con) and DTT-augmented phosphorylated TrkAIII levels (pTrkAIII) in extracts from stable TrkAIII SH-SY5Y transfectants (5 μg). β-actin levels are shown as loading controls (m = markers). In all experiments, DTT was used at a concentration of 5 mM.

To further investigate the relationship between ER stress and alternative TrkAIII splicing, TrkAIII expression and Xbp-1 splicing were assessed in *BRAF (V600E)*-mutated A375 melanoma cells following treatment with the reducing agent DTT, which nowadays is widely used as a rapid inducer of ER stress [46]. DTT treatment resulted in unconventional Xbp-1 splicing, confirming UPR activation [44,45,46] and significantly enhanced TrkAIII mRNA expression, relative to 18S rRNA, at 6 h (*p* = 0.0114, df = 4) and 12 h (*p* = 0.007, df = 4) (Figure 4A).

In indirect IF analyses, endogenous non-phosphorylated TrkA expressed in A375 cells was phosphorylated following a 6 h treatment with DTT in the absence but not in the presence of lestaurtinib (Figure 4C). In Western blots, non-phosphorylated 130–140 kDa TrkA, detected in untreated A375 cell extracts, was converted into a phosphorylated ≈100 kDa TrkA isoform, following a 6 h DTT treatment in the absence but not presence of lestaurtinib, that closely resembled constitutively phosphorylated TrkAIII in extracts from stable TrkAIII-transfected SH-SY5Y cells, which was also augmented by DTT (Figure 4D).

These data demonstrate that UPR activation by the reducing agent DTT promotes TrkAIII mRNA expression and alters TrkA protein expression, resulting in the expression of a phosphorylated TrkA isoform, consistent with TrkAIII, in A375 melanoma cells.

### 3.5. Transient Fully Spliced TrkA and TrkAIII A375 Transfectants

Fully spliced TrkA and alternatively spliced TrkAIII behaviour were compared under normal conditions and following DTT treatment, by transient transfection of A375 cells with fully spliced TrkA and TrkAIII pcDNA3.1 mammalian expression vectors [14].

**Figure 5:**
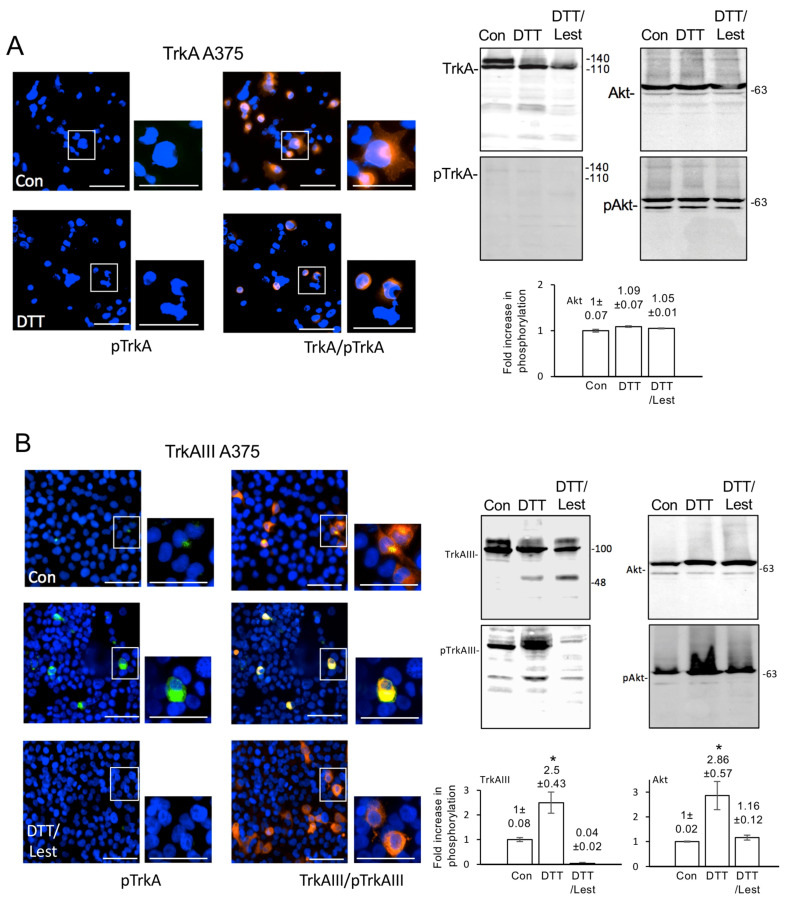

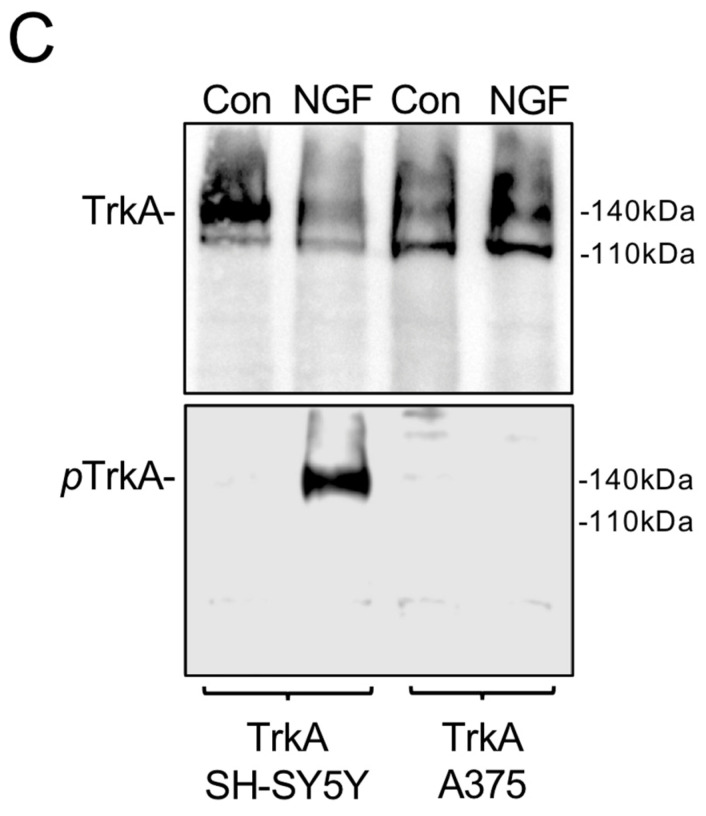
DTT augments TrkAIII and Akt phosphorylation in A375 transfectants. (**A**) Micrographs demonstrating immunoreactivity to mouse monoclonal anti-TrkA (B3) antibody (orange) but not to rabbit monoclonal anti-pY490-phosphorylated TrkA antibody (green, pTrkA) in untreated DTT-treated transient fully spliced TrkA A375 transfectants (bar = 50 μm). The Western blots demonstrate changes in fully spliced TrkA maturation, without phosphorylation (pTrkA), and no change in Akt and phosphorylated Akt (pAkt) levels in fully spliced TrkA A375 extracts, following DTT treatment in the absence (DTT) and presence of lestaurtinib (DTT/Lest) (50 μg loads). The histogram demonstrates the lack of DTT effect on Akt phosphorylation following DTT treatment in the absence or presence of lestaurtinib, in fully spliced TrkA A375 extracts (50 μg loads), expressed as mean ± s.e. fold increase in phosphorylation compared to untreated controls (1 fold), in 3 independent experiments; (**B**) micrographs demonstrating TrkAIII immunoreactivity to mouse monoclonal anti-TrkA (B3) antibody (orange), phosphorylated TrkAIII immunoreactivity to rabbit monoclonal anti-pY490-phosphorylated TrkA antibody (green, pTrkA), overlapping immunoreactivity (yellow) and DAPI-stained nuclei (blue) in untreated TrkAIII A375 transfectants (upper panels) and augmented phosphorylated TrkAIII immunoreactivity following DTT treatment (middle panels, green/yellow) and absence of phosphorylated TrkAIII immunoreactivity (pTrkAIII) following co-treatment with DTT and lestaurtinib (DTT/Lest) (lower panels) (bar = 50 μm). The Western blots demonstrate augmented phosphorylated TrkAIII (pTrkAIII) levels in TrkAIII A375 transfectants following DTT treatment in the absence (DTT) but not in the presence of lestaurtinib (DTT/Lest), compared to untreated controls (Con) (50 μg loads) (left panels), and augmented Akt phosphorylation (pAkt) in TrkAIII A375 transfectants treated with DTT in the absence (DTT) but not in the presence of lestaurtinib (DTT/Lest), compared to untreated controls (Con) (50 μg loads). Histograms display significant increases (*) in TrkAIII and Akt phosphorylation following DTT treatment in the absence (DTT) but not presence of lestaurtinib (DTT/Lest), expressed as the densitometric mean ± s.e. fold increase in phosphorylation, compared to untreated controls (1 fold), in 3 independent experiments. In all experiments, DTT was used at a concentration of 50 mM and lestaurtinib at 100 nM, and the treatment duration was 6 h. Magnifications of boxed areas are also provided to the left of each micrograph to determine expression in more detail. (**C**) Western blots demonstrating NGF (100 ng for 15 min) induction of fully spliced TrkA phosphorylation in SH-SY5Y transfectants (20 μg loads) but not in fully spliced TrkA A375 transfectants (50 μg loads).

In Western blots, fully spliced TrkA expressed in A375 transfectants exhibited impaired gp 140 kDa maturation and was not activated by exogenous NGF (Figure 5A,C). In contrast, fully spliced TrkA in SH-SY5Y transfectants exhibited rapid gp 140 kDa maturation and NGF-induced activation (this study and [23,47]) (Figure 5C).

In contrast to fully spliced TrkA dysfunction in A375 cells, TrkAIII behaviour was similar in A375 and SH-SY5Y cells. In both cell types, TrkAIII was expressed as a 100 kDa constitutively phosphorylated intracellular protein, the phosphorylation of which was significantly augmented by DTT in A375 cells (*p* = 0.008, df = 4) and in SH-SY5Y transfectants (Figure 4 and [23]). The Trk inhibitor lestaurtinib reduced DTT-augmented TrkAIII phosphorylation in A375 cells to below constitutive TrkAIII phosphorylation levels in untreated A375 controls (*p* = 0.002 df = 4) (Figure 5B).

Considering reports that TrkAIII signals through the IP3K/Akt pathway in NB cells (14), the effects of DTT treatment on Akt phosphorylation were also assessed in fully spliced TrkA and TrkAIII A375 transfectants. In Western blots, DTT significantly augmented constitutive Akt phosphorylation in TrkAIII (*p* = 0.002, df = 4) but not fully spliced TrkA A375 transfectants, and lestaurtinib reduced DTT-augmented Akt phosphorylation in TrkAIII A375 transfectants to constitutive levels. In contrast, lestaurtinib did not reduce constitutive Akt phosphorylation in fully spliced TrkA A375 transfectants (Figure 5A,B).

These data demonstrate that DTT induces TrkAIII but not fully spliced TrkA phosphorylation in transient A375 melanoma cell transfectants. The data also demonstrate that DTT-induced TrkAIII phosphorylation is prevented by lestaurtinib, and that fully spliced TrkA is not activated by NGF in transient A375 transfectants.

### 3.6. TrkAIII Enhances A375 Resistance to DTT-Induced Death

**Figure 6:**
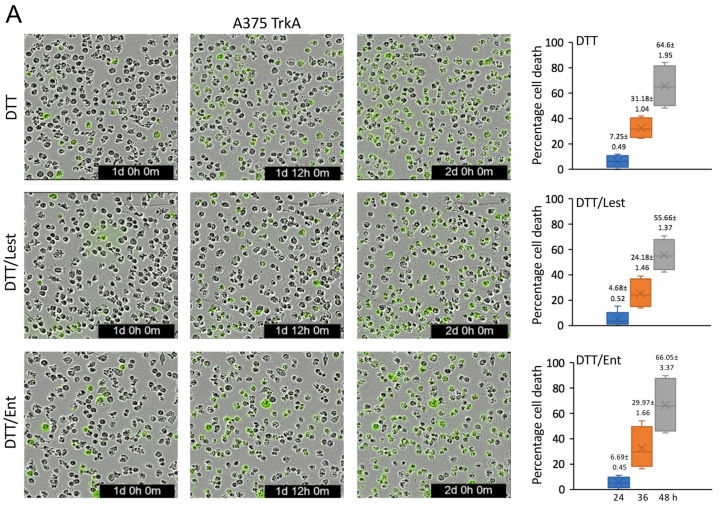

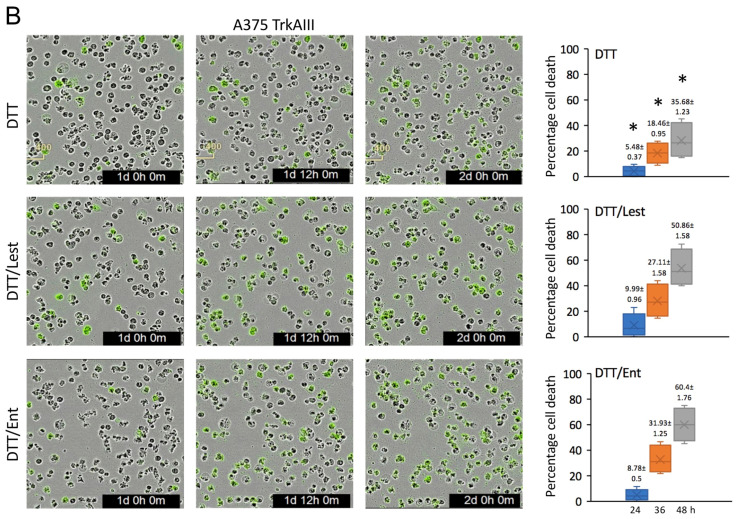
Resistance to DTT-induced death in A375 cells conferred by TrkAIII is prevented by lestaurtinib and entrectinib. Phase contrast micrographs demonstrating enhanced nuclear green dye uptake in fully spliced TrkA (**A**) compared to TrkAIII (**B**) A375 transfectants, following DTT treatment (5 mM) for 24, 36 and 48 h, and the re-sensitisation of TrkAIII but not fully spliced TrkA A375 transfectants to DTT-induced nuclear Incucyte Green Dye uptake in the presence of lestaurtinib (100 nM) and entrectinib (100 nM). Box plots demonstrate these changes as significant (*) reductions in DTT-induced death in TrkAIII A375 transfectants at 24, 36 and 48 h compared to fully spliced TrkA A375 transfectants, in the absence (DTT) but not presence of 100 nM lestaurtinib (DTT/Lest) or 100 nM entrectinib (DTT/Ent). Results are expressed as mean ± s.e. percentage cell death in 3 independent experiments, performed in duplicate.

In Incucyte cytotoxicity assays, the percentage of cells exhibiting nuclear Cytotox Green Dye accumulation, as an index of cell death, was compared in fully spliced TrkA and TrkAIII A375 transfectants, over a 48 h time course of DTT treatment. DTT did not induce significant nuclear uptake of Incucyte Green Dye prior to 24 h in either fully spliced TrkA or TrkAIII A375 transfectants. At 24 h, 7.25% of fully spliced TrkA A375 transfectants exhibited nuclear Incucyte Green Dye uptake, which increased to 31.18% by 36 h and 64.6% by 48 h (Figure 6A). In TrkAIII A375 transfectants, nuclear Incucyte Green Dye uptake was significantly reduced to 5.48% at 24 h (*p* = 0.0163, df = 10), to 18.46% at 36 h (*p* < 0.0001, df = 10) and to 35.68% by 48 h (*p* < 0.0001, df = 10), compared to fully spliced TrkA A375 transfectants (Figure 6B). DTT co-treatment with lestaurtinib or entrectinib did not significantly alter nuclear Incucyte Green Dye uptake in fully spliced TrkA A375 transfectants (Figure 6A) but significantly enhanced nuclear Incucyte Green Dye uptake in TrkAIII A375 transfectants at 24, 36 and 48 h to levels that did not significantly differ from those of fully spliced TrkA A375 transfectants (Figure 6B).

These data demonstrate that TrkAIII but not fully spliced TrkA confers enhanced resistance to DTT-induced cell death in transient A375 transfectants, and that this effect is prevented by the Trk inhibitors lestaurtinib and entrectinib.

## 4. Discussion

In this first report of alternative TrkA mRNA splicing in CMMs and TrkA splice variant behaviour in *BRAF(V600E)*-mutated A375 melanoma cells, we demonstrate a possible causal relationship between reductive stress, UPR activation, alternative TrkA splicing and TrkAIII expression and activation, consistent with a targetable, pro-survival, oncogenic role for TrkAIII in metastatic CMM progression. We characterise TrkAIII as the only splice variant expressed in CMMs with the potential to produce an oncogenic substitute for fully spliced TrkA. We report that TrkAIII expression was more frequently detected in metastatic *BRAF* wild-type and *BRAF*-mutated CMMs, predominated over fully spliced TrkA in ≈50% of metastatic CMMs, was associated with UPR activation and intracellular phosphorylated TrkA isoform immunoreactivity, and phosphorylated TrkA species, resembling TrkAIII, were detected in metastatic CMM extracts. We also show that reductive stress activates the UPR and promotes TrkAIII mRNA expression in *BRAF(V600E)*-mutated A375 melanoma cells and, in transient TrkAIII A375 transfectants, promotes TrkAIII and Akt phosphorylation, resulting in increased resistance to reductive-stress-induced death, which is prevented by the Trk inhibitors lestaurtinib and entrectinib. Last but not least, we report that fully spliced TrkA is dysfunctional in A375 melanoma cells.

ER stress involvement in alternative TrkA mRNA splicing in CMMs was suggested by association with unconventional Xbp-1 splicing, indicating UPR activation [44,45]. This extends previous reports of UPR activation in CMM [29,30,31,32], and is supported by the observation that reductive stress induced by DTT activated the UPR and promoted TrkAIII mRNA expression in A375 melanoma cells (this study), and by previous reports that agents that induce ER stress and activate the UPR promote alternative TrkAIII splicing in SH-SY5Y NB cells [14,15,25]. Alternative TrkA mRNA splicing in CMM may also result from mutations in the spliceosome component SF3B1 shown to promote exon skipping [48] but is unlikely to depend upon *BRAF* mutation, as alternative TrkA mRNA splicing was detected in both *BRAF wild-type* and *BRAF-mutated* CMMs.

Within the context of experiments demonstrating a pro-survival role for TrkAIII in A375 cells combined with fully spliced TrkA dysfunction, the pattern of alternative TrkA mRNA splicing detected in CMMs (see Figure 2B) not only confirms that TrkA splicing is significantly de-regulated but also suggests a potential oncogenic role for TrkAIII in a significant CMM subpopulation. This is supported by the fact that TrkAIII was the only splice variant expressed in CMMs with oncogenic potential, and it was more frequently detected in metastatic CMMs, in which it predominated over fully spliced TrkA mRNA expression in ≈50%. TrkAIII mRNA expression, furthermore, was invariably associated with intracellular phosphorylated TrkA isoform immunoreactivity, and phosphorylated species resembling TrkAIII were detected in metastatic CMMs. Moreover, TrkAIII expression and activation in *BRAF(V600E)*-mutated A375 melanoma cells were promoted by DTT-induced reductive stress, resulting in enhanced Akt phosphorylation and increased resistance to DTT-induced death. These effects were confirmed as TrkAIII-dependent using the Trk inhibitor lestaurtinib [37], which prevented constitutive and DTT-enhanced TrkAIII phosphorylation and prevented DTT-enhanced but not constitutive Akt phosphorylation in TrkAIII A375 transfectants, and re-sensitised TrkAIII A375 transfectants to DTT-induced death. A similar effect was observed using the Trk inhibitor entrectinib [38], which also re-sensitised TrkAIII A375 transfectants to DTT-induced death. Evidence linking Akt phosphorylation to TrkAIII phosphorylation and augmented stress resistance in A375 cells extends previous reports that TrkAIII signals through IP3K/Akt, resulting in enhanced resistance to DTT-induced death in SH-SY5Y NB cells [14,15]. Although not studied in A375 cells, in SH-SY5Y cells this effect is prevented by dantrolene, implicating ryanodine receptors and ER Ca^2+^ release [23,49,50,51]. This suggests a role for stress-induced Ca^2+^ transients in promoting alternative TrkAIII splicing and TrkAIII activation, and implicates a role for TrkAIII in mitigating cytoplasmic Ca^2+^ cytotoxicity under conditions of reductive ER stress.

The detection of intracellular TrkA and phosphorylated TrkA isoform immunoreactivity in CMMs in the present study extends previous reports that enhanced TrkA expression and intracellular phosphorylated TrkA immunoreactivity are associated with poor prognosis in CMM [9,10], but contrasts with a recent report that TrkA immunoreactivity is not associated with CMM progression [13]. This contradiction can be explained by the rabbit anti-human TrkA monoclonal anti-TrkA antibody (14G6, CS-2508, Cell Signaling) used in the latter study [13], raised against a synthetic peptide surrounding TrkA arginine 220 within the D4 domain, which would detect fully spliced TrkA but not TrkAIII [14]. The lack of immunoreactivity in metastatic CMMs reported using this antibody [13] suggests that the immunoreactivity detected in metastatic CMMs using antibodies that recognise non-phosphorylated and phosphorylated forms of both fully spliced TrkA and TrkAIII (this study and [9,10]) was in fact due to TrkAIII, which is supported by the detection of ≈100 kDa phosphorylated TrkAIII-like species in TrkAIII mRNA-expressing metastatic CMMs.

The alternative TrkA splice variants detected in CMMs were identical to those recently reported in MCPyV-positive Merkel cell carcinomas [18,19], extending the potential oncogenic role of TrkAIII, originally reported in human NB [14], to MCPyV-positive Merkel cell carcinoma [18,19] and CMM (this study). In SH-SY5Y NB cells, alternative TrkAIII splicing is also promoted by SV40 poliomavirus large T-antigen expression [15], suggesting that TrkAIII expression in MCPyV-positive Merkel cell carcinomas may be promoted by MCPyV large T antigen [18,19]. Here, we show that reductive stress promotes TrkAIII expression and activation in melanoma cells, extending reports that TrkAIII mRNA expression is promoted by hypoxia [14,25]. As hypoxia promotes reductive stress, which in turn promotes tumour progression [27,28], conditions that promote reductive stress in CMMs may underpin alternative TrkA splicing, TrkAIII expression and TrkAIII activation.

In contrast to TrkAIII, fully spliced TrkA was dysfunctional in A375 cells, exhibited impaired gp140 kDa maturation and was not activated by exogenous NGF. This contrasts with fully spliced TrkA behaviour in SH-SY5Y cells, which exhibits rapid gp140 kDa maturation and NGF-induced activation (this study and [14,23,47,52]). This dysfunction may reflect reduced GCNT2 glycosylation branching enzyme activity, which in A375 cells impairs insulin growth factor receptor maturation and function and in CMMs is associated with metastatic progression [53]. This unveils a novel mechanism, in addition to alternative *TrkA* splicing, for abrogating melanoma-suppressing fully spliced TrkA potential [7,13].

We propose, therefore, that alternative TrkA splicing, resulting in TrkAIII expression and activation, adds a novel targetable pro-survival oncogenic mechanism to UPR activation [29,30] and low melanoma-inducing transcription factor/anexelecto receptor tyrosine kinase (MITF/Axl) ratios [54] in CMM, which can be blocked by the Trk inhibitors lestaurtinib and entrectinib.

## 5. Conclusions

This first study of alternative TrkA splicing in CMM and in *BRAF(V600E)*-mutated A375 melanoma cells suggests a causal link between reductive stress, UPR activation, alternative TrkA splicing, TrkAIII expression and intracellular activation and metastatic CMM, consistent with a stress-induced, oncogenic, pro-survival role for TrkAIII in CMM progression. Furthermore, fully spliced TrkA dysfunction in A375 cells unveils a novel mechanism, other than alternative TrkA splicing, for abrogating TrkA melanoma-suppressing potential. Our observations help to explain contradicting reports of TrkA involvement in CMM, characterise reductive stress as a promoter of TrkAIII expression and oncogenic activity and identify lestaurtinib and entrectinib as therapeutic inhibitors of this novel potential oncogenic mechanism in CMM.

## Figures and Tables

**Table 1:** Summary of associations between TrkA mRNA expression, alternative TrkA mRNA splicing and intracellular TrkA and phosphorylated TrkA IF immunoreactivity in primary and metastatic CMMs. Levels of TrkA relative to 18S rRNA RT-PCR products are displayed as: high, moderate (Mod), low or not detected (No). TrkA exon 1–8 alternative splicing is displayed as detected (Yes) or not detected (No). Relative levels of TrkAIII exon 1–8 (TrkAIII (ex 1–8) RT-PCR) and TrkAIII exon 5/8 splice junction RT-PCR products in snap-frozen (TrkAIII (ex 5/8) RT-PCR) and formalin-fixed paraffin-embedded tissues (TrkAIII (ex 5/8) RT-PCR FFPE), and TrkA (TrkA IF) and phosphorylated TrkA immunoreactivity (pTrkA IF), are displayed as high to low (+++ to +), very low (±), or not detected (-). Unconventional Xbp-1 splicing is displayed as detected (Yes) or not detected (No) (see Supplemental Figures S1–S4 for CMM IF micrographs and RT-PCRs). Only formalin-fixed paraffin-embedded (FFPE) tissues were available for patients P.28a/b, P.29a/b and P.30a/b. ND refers to observations that were not carried out due to limited RNA and tissues.

PRIMARY CMMs.													
Patient	P.1	P.2	P.3	P.4	P.5		P.6	P.7	P.8	P.28a FFPE	P.29a FFPE	P.30a FFPE	NS
TrkA relative to 18S rRNA	High	Mod/High	High	High	High		Mod/ Low	Low	High	ND	ND	ND	High
TrkA exon 1-8 Alt. splicing	Yes	Yes	No	Yes	Yes		No	Yes	No	ND	ND	ND	No
TrkAIII exon 1–8 RT-PCR	++	+	-	+	-		-	-	-	ND	ND	ND	-
TrkAIII exon 5–8 RT-PCR	+++	+	±	-	-		-	-	ND	ND	ND	ND	-
TrkAIII exon 5–8 RT-PCR FFPE	+	+	±	±	-		-	-	ND	+	+	+	-
TrkA IF	++±	+++	+	+++	++		++	-	ND	+++	+++	+++	+++
pTrkA IF	++	+++	-	+±	-		±	-	ND	+++	++	+++	-
Xbp1 splicing	Yes	Yes	Yes	Yes	ND		ND	ND	ND	Yes	Yes	Yes	No
**METASTATIC CMMs**													
**Patient**	**P.9**	**P.10**	**P.11**		**P.12**	**P.13**	**P.14**	**P.15**	**P.16**	**P.17**	**P.18**	**P.19**	**P.20**
TrkA relative to 18S rRNA	Mod/ High	High	Mod/ Low		Mod/ Low	Mod/ High	Mod/ Low	Low	Mod/ High	Mod/ High	High	High	High
TrkA exon 1-8 Alt splicing	Yes	Yes	Yes		Yes	Yes	Yes	Yes	Yes	Yes	Yes	Yes	Yes
TrkAIII exon 1–8 RT-PCR	+++	+++	+++		++	+++	+	±	+++	+++	+	+	±
TrkAIII exon 5–8 RT-PCR	±	+	++		+	++±	+	+	ND	ND	+	+	+
TrkAIII exon 5–8 RT-PCR FFPE	+	+	+		+	+	+	+	ND	ND	+	+	-
TrkA IF	++±	+++	++±		++±	++	++±	++	ND	ND	++±	++±	++
pTrkA IF	++	+++	++±		+±	++	±	++	ND	ND	++±	++±	+±
Xbp1 splicing	Yes	Yes	Yes		Yes	Yes	Yes	Yes	ND	ND	Yes	Yes	Yes
**Patient**	**P:21**	**P.22**	**P.23**		**P.24**	**P.25**	**P.26**	**P.27**	**P.28b FFPE**		**P.29b FFPE**		**P.30b FFPE**
TrkA relative to 18S rRNA	High	High	Low		Low	Mod/ High	No	No	ND		ND		ND
TrkA exon 1-8 Alt splicing	Yes	No	No		No	No	No	No	ND		ND		ND
TrkAIII exon 1–8 RT-PCR	-	-	-		-	-	-	-	ND		ND		ND
TrkAIII exon 5–8 RT-PCR	-	±	-		-	ND	±	-	ND		ND		ND
TrkAIII exon 5–8 RT-PCR FFPE	±	-	+		-	ND	-	-	+		+		±
TrkA IF	++±	+	+++		-	ND	-	-	+++		+++		+++
pTrkA IF	++±	±	+++		-	ND	-	-	+++		++		+++
Xbp1 splicing	Yes	Yes	Yes		Yes	ND	Yes	Yes	Yes		Yes		Yes

## Data Availability

The data sets used and/or analysed during this study are either included in this published article or are available from the corresponding author, upon reasonable request.

## Supplementary Materials

The following supporting information can be downloaded at: https://www.mdpi.com/article/10.3390/cells12020237/s1, Table S1: Patients. Details of the 30 CMM patient cohort, including: gender, age, *BRAF*-mutation status (ND = not done), tumor type (primary or metastatic) and stage. Figure S1: Alternative TrkA splicing and intracellular expression of tyrosine phosphorylated TrkA isoforms in primary CMMs. Ethidium bromide-stained agarose gels demonstrating 18S rRNA, TrkA-specific, TrkAIII-specific and alternative TrkA splice variant and Xbp1 un-spliced (u) and spliced (s) RT-PCR products, in RNAs from 7 fresh primary CMMs and in uninvolved skin samples (NS1 and NS2) (m = DNA markers), plus micrographs demonstrating variable levels of TrkA and phosphorylated TrkA (pTrkA) IF immunoreactivity in each primary CMM and 2 uninvolved skin sample (NS1 and NS2); Figure S2: Alternative TrkA splicing and intracellular expression of tyrosine phosphorylated TrkA isoforms in metastatic CMMs. Ethidium bromide-stained agarose gels demonstrating 18S rRNA, TrkA-specific, TrkAIII-specific and alternative TrkA splice variant plus un-spliced (u) and spliced (s) Xbp1 RT-PCR products, in RNAs from 16 fresh CMM metastases and uninvolved skin (m = DNA markers), plus micrographs demonstrating variable levels of TrkA and phosphorylated TrkA IF immunoreactivity in each CMM metastasis. Normal skin (NS) and CMM tissues (CMM) were also incubated with secondary antibodies alone (secondary antibody), in order to confirm the specificity of primary antibodies; Figure S3: TrkAIII mRNA and intracellular tyrosine phosphorylated TrkA expression in paired primary and metastatic formalin-fixed paraffin-embedded (FFPE) CMM tissues. Micrographs demonstrating TrkA and phosphorylated TrkA IF immunoreactivity, and ethidium bromide-stained agarose gels demonstrating 18s rRNA, TrkA-specific, TrkAIII-specific and Xbp1 un-spliced (u) and spliced (s) RT-PCR products in paired primary (a) and metastatic (b) CMM FFPE tissues (patients P.28-30); Figure S4: Alternative TrkA splicing in additional primary (P.8) and metastatic CMMs (P.16, P.17 and P.25). RT-PCRs demonstrating products corresponding to fully spliced TrkA (fsTrkA), TrkAIII, Δ ex 2-7 TrkA and 18S rRNA, generated using TrkA exon 1-8 and 18S rRNA primers, in P.8’s primary CMM and P.16’s, P.17’s and P.25’s metastatic CMMs.
