# Supplementary material for: The Alternative TrkAIII Splice Variant, a Targetable Oncogenic Participant in Human Cutaneous Malignant Melanoma
Source: Cells. 2023 Jan 5;12(2):237. doi: 10.3390/cells12020237 (PMC9856487; doi:10.3390/cells12020237)
Supplement: Supplementary file 1 [file cells-12-00237-s001.zip › cells-2111175-supplementary.pdf]

Table S1. Patients. Details of the 30 CMM patient cohort, including: gender, age, *BRAF*-mutation status (ND = not done), tumor type (primary or metastatic) and stage.

Patient	Gender/Age	*BRAF*-status	Tumor Type	Stage
P.1	M/62	*V600E* mutated	Primary	IIIC
P.2	F/84	Wild Type	Primary	IIID
P.3	F/39	Wild Type	Primary	IA
P.4	F/83	Wild Type	Primary	IIB
P.5	F/81	Wild Type	Primary	IA
P.6	M/48	Wild Type	Primary	IIB
P.7	M/72	*V600E* mutated	Primary	IIIC
P.8	M/73	ND	Primary	IIIC
P.9	F/59	Wild Type	Metastasis	IIIB
P.10	F/78	Wild Type	Metastasis	IIIC
P.11	M/53	Wild Type	Metastasis	IIIC
P.12	F/78	Wild Type	Metastasis	IIA
P.13	M/69	*V600E* mutated	Metastasis	IIIC
P.14	M/43	*V600E* mutated	Metastasis	IIIC
P.15	M/53	*V600E* mutated	Metastasis	IIIC
P.16	M/80	*V600E* mutated	Metastasis	IIID
P.17	M/87	Wild Type	Metastasis	IIIC
P.18	M/89	Wild Type	Metastasis	IIIC
P.19	M/81	Wild Type	Metastasis	IIIC
P.20	F/78	ND	Metastasis	IIID
P.21	F/74	*V600E* mutated	Metastasis	IIIC
P.22	M/52	Wild Type	Metastasis	IIIC
P.23	M/64	*V600E* mutated	Metastasis	IIID
P.24	M/81	Wild Type	Metastasis	IIID
P.25	M/95	Wild Type	Metastasis	IV
P.26	M/56	*V600E* mutated	Metastasis	IV
P.27	M/84	Wild Type	Metastasis	IV
P.28	F/77	Wild Type	Primary and Metastasis	IV
P.29	M/70	*V600K* mutated	Primary and Metastasis	III
P.30	F/35	*V600E* mutated	Primary and Metastasis	III

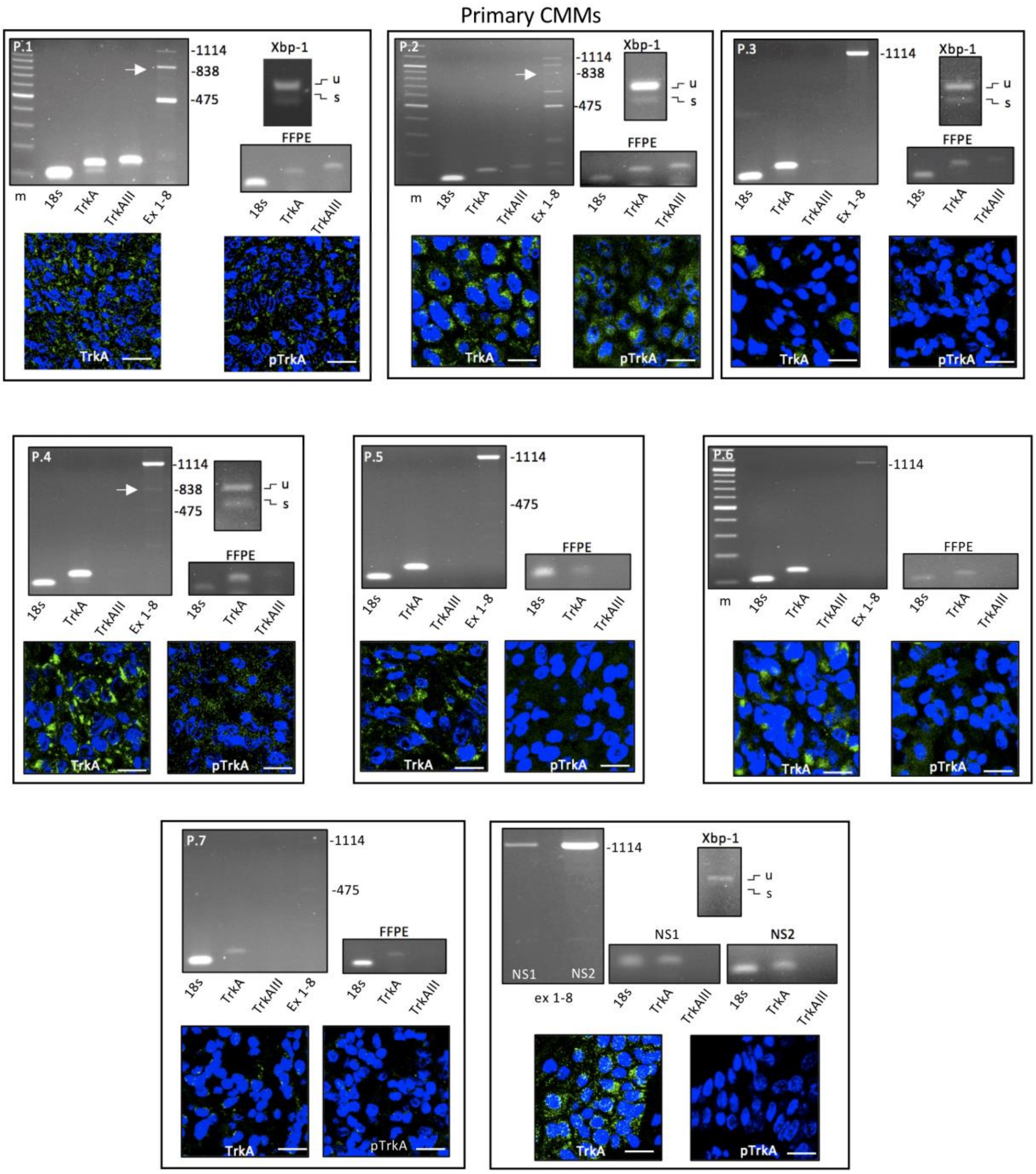

Figure S1. Alternative TrkA splicing and intracellular expression of tyrosine phosphorylated TrkA isoforms in primary CMMs. Ethidium bromide-stained agarose gels demonstrating 18S rRNA, TrkA-specific, TrkAIII-specific and alternative TrkA splice variant and Xbp1 un-spliced (u) and spliced (s) RT-PCR products, in RNAs from 7 fresh primary CMMs and in uninvolved skin samples (NS1 and NS2) (m = DNA markers), plus micrographs demonstrating variable levels of TrkA and phosphorylated TrkA (pTrkA) IF immunoreactivity in each primary CMM and 2 uninvolved skin sample (NS1 and NS2).

Metastatic CMMs

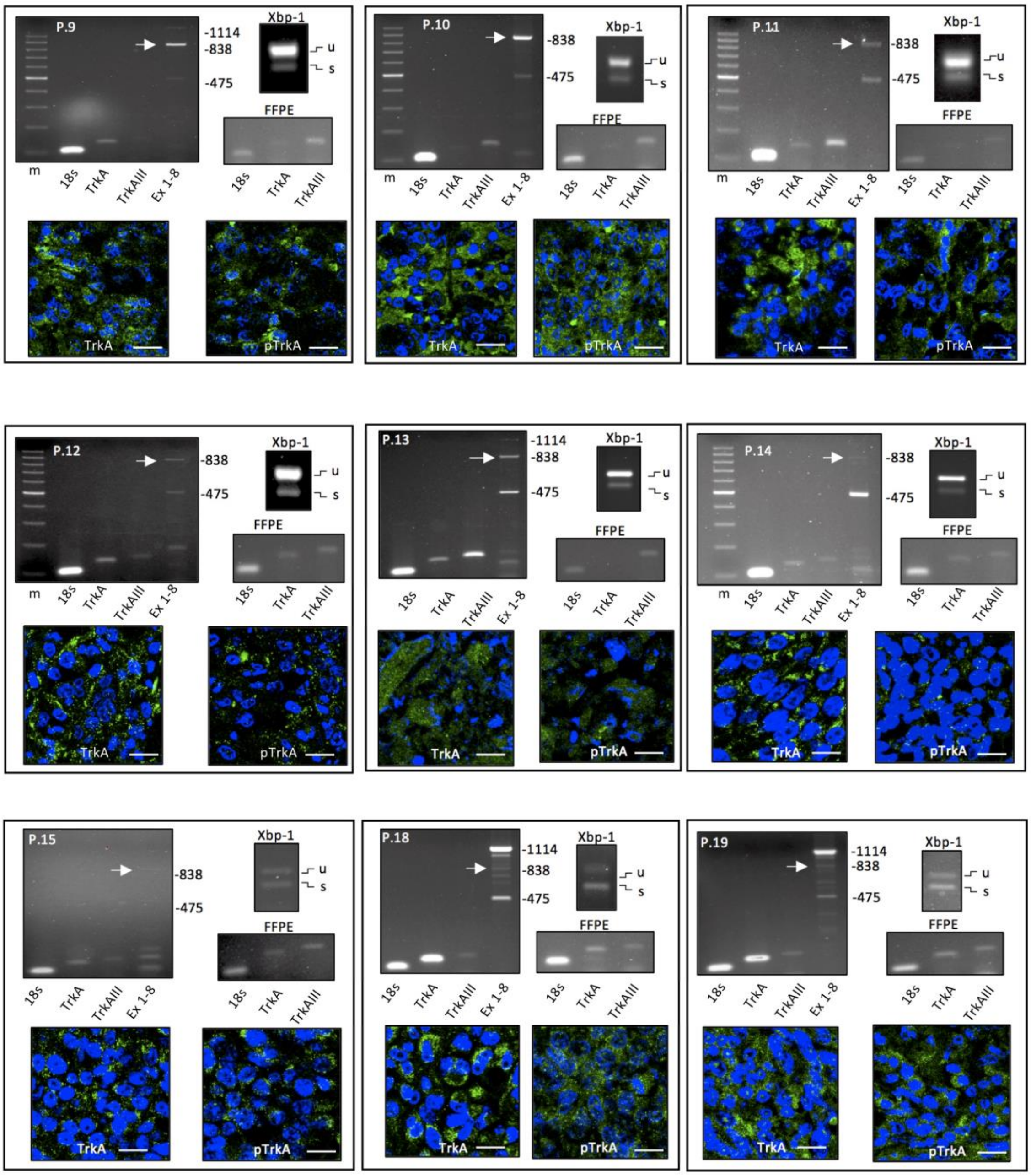

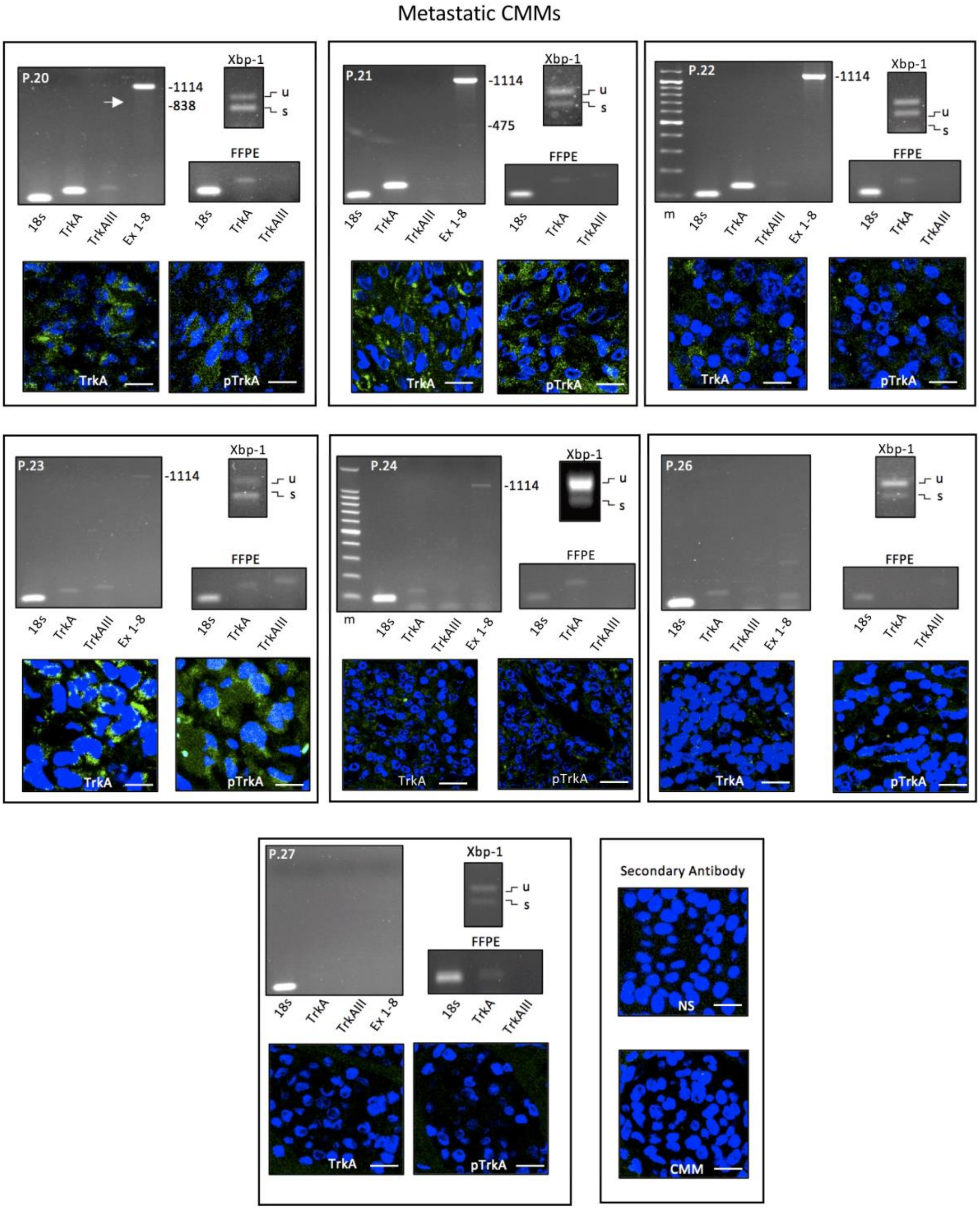

Figure S2. Alternative TrkA splicing and intracellular expression of tyrosine phosphorylated TrkA isoforms in metastatic CMMs. Ethidium bromide-stained agarose gels demonstrating 18S rRNA, TrkA-specific, TrkAIII-specific and alternative TrkA splice variant plus un-spliced (u) and spliced (s) Xbp1 RT-PCR products, in RNAs from 16 fresh CMM metastases and uninvolved skin (m = DNA markers), plus micrographs demonstrating variable levels of TrkA and phosphorylated TrkA IF immunoreactivity in each CMM metastasis. Normal skin (NS) and CMM tissues (CMM) were also incubated with secondary antibodies alone (secondary antibody), in order to confirm the specificity of primary antibodies.

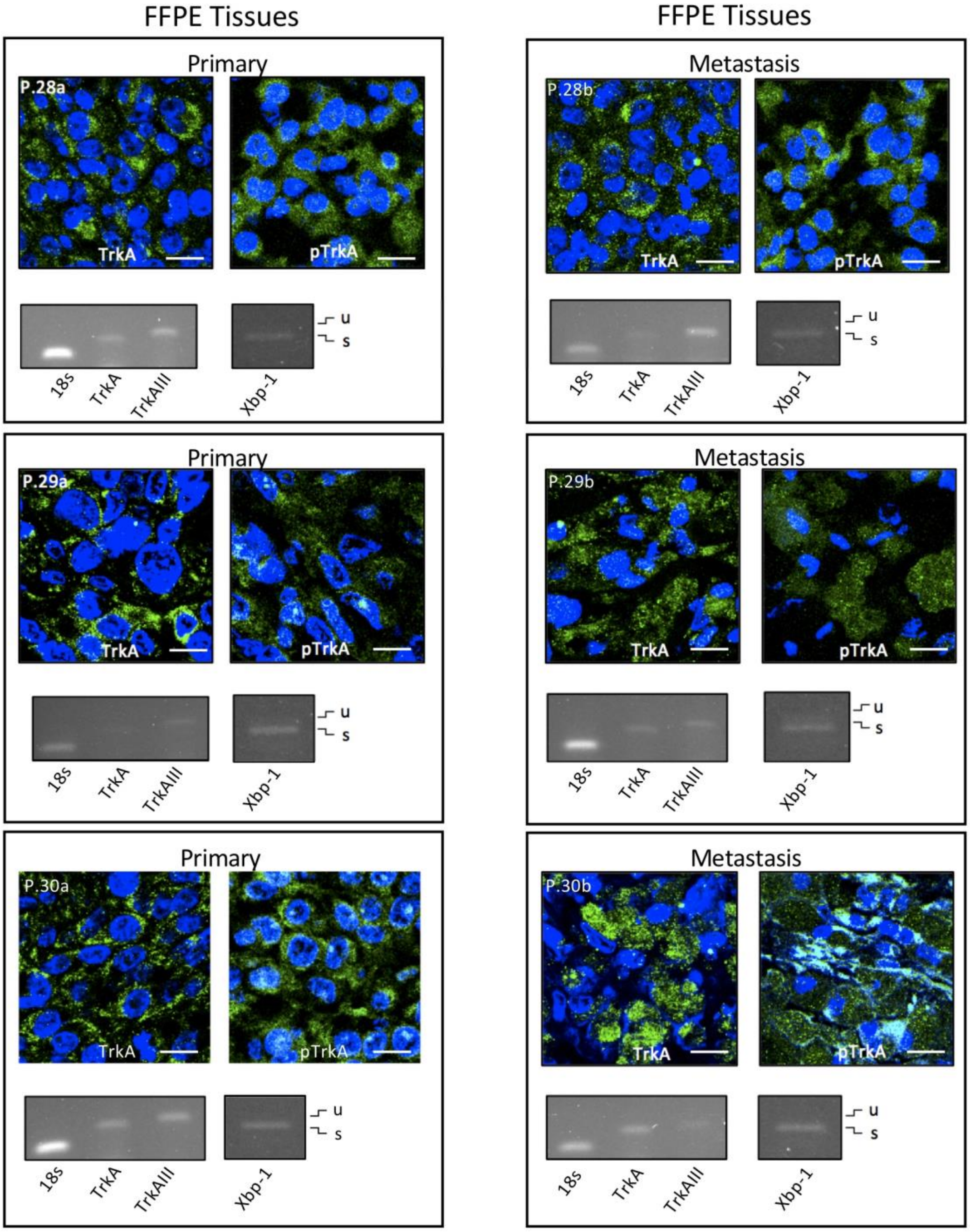

Figure S3. TrkAIII mRNA and intracellular tyrosine phosphorylated TrkA expression in paired primary and metastatic formalin-fixed paraffin-embedded (FFPE) CMM tissues. Micrographs demonstrating TrkA and phosphorylated TrkA IF immunoreactivity, and ethidium bromide-stained agarose gels demonstrating 18s rRNA, TrkA-specific, TrkAIII-specific and Xbp1 un-spliced (u) and spliced (s) RT-PCR products in paired primary (a) and metastatic (b) CMM FFPE tissues (patients P.28-30).

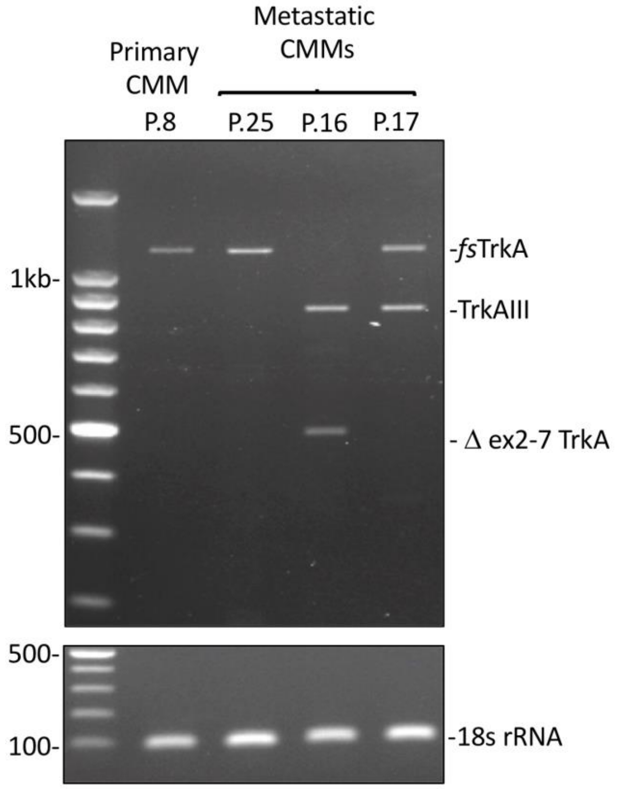

Figure S4. Alternative TrkA splicing in additional primary (P.8) and metastatic CMMs (P.16, P.17 and P.25). RT-PCRs demonstrating products corresponding to fully spliced TrkA (fsTrkA), TrkAIII, Δ ex 2-7 TrkA and 18S rRNA, generated using TrkA exon 1-8 and 18S rRNA primers, in P.8's primary CMM and P.16's, P.17's and P.25's metastatic CMMs.
